# Biochemical phosphates observed using hyperpolarized ^31^P in physiological aqueous solutions

**DOI:** 10.1038/s41467-017-00364-3

**Published:** 2017-08-24

**Authors:** Atara Nardi-Schreiber, Ayelet Gamliel, Talia Harris, Gal Sapir, Jacob Sosna, J. Moshe Gomori, Rachel Katz-Brull

**Affiliations:** Department of Radiology, Hadassah-Hebrew University Medical Center, Jerusalem, Israel

## Abstract

The dissolution-dynamic nuclear polarization technology had previously enabled nuclear magnetic resonance detection of various nuclei in a hyperpolarized state. Here, we show the hyperpolarization of ^31^P nuclei in important biological phosphates (inorganic phosphate and phosphocreatine) in aqueous solutions. The hyperpolarized inorganic phosphate showed an enhancement factor >11,000 (at 5.8 T, 9.3% polarization) in D_2_O (T_1_ 29.4 s). Deuteration and the solution composition and pH all affected the lifetime of the hyperpolarized state. This capability opens up avenues for real-time monitoring of phosphate metabolism, distribution, and pH sensing in the live body without ionizing radiation. Immediate changes in the microenvironment pH have been detected here in a cell-free system via the chemical shift of hyperpolarized inorganic phosphate. Because the ^31^P nucleus is 100% naturally abundant, future studies on hyperpolarized phosphates will not require expensive isotope labeling as is usually required for hyperpolarization of other substrates.

## Abstract (web summary)

Real-time monitoring of phosphate metabolism and distribution in the live body without ionizing radiation is highly desirable. Here, the authors show dissolution-dynamic nuclear polarization technology can enable nuclear magnetic resonance detection of hyperpolarized ^31^P of important biological phosphates in aqueous solutions.

## Introduction

Recent advances in the dissolution-dynamic nuclear polarization (d-DNP) and para-hydrogen-induced polarization (PHIP)-based hyperpolarization techniques have revolutionized the field of liquid-state nuclear magnetic resonance (NMR) spectroscopy. By providing signal enhancements of up to four orders of magnitude, such methods enable the detection of small amounts of materials as well as detections made on very short time scales. The d-DNP technology, pioneered by Ardenkjaer-Larsen et al.^1^, has been predominantly focused on metabolic work using hyperpolarized ^13^C sites^2^. A few studies looked at metabolites labeled with ^15^N^3, 4^. In the field of contrast material, hyperpolarized yttrium showed beneficial relaxation properties^5, 6^ and water protons were also hyperpolarized^7–10^. d-DNP studies with ^107,109^Ag^11^, ^6^Li^12, 13^, ^29^Si^14^, and ^19^F^15^ have been reported as well. However, to date, to the best of our knowledge, the phosphorus nucleus (^31^P), which is 100% naturally abundant and plays an important role in chemistry and life, had not been seen in a d-DNP-driven hyperpolarized state in aqueous solutions. One study reported the ability to hyperpolarize the ^31^P nuclei of adenosine triphosphate (ATP) in the solid state using DNP^16^. However, this study was not followed-up by detection of this hyperpolarized state in solution. Since the T_1_ of the ^31^P nuclei of ATP in aqueous solutions is very short, on the order of 1 s^17^, it would be highly challenging to visualize ATP in a hyperpolarized state in solution. In our hands, this was indeed impossible (data not shown).

PHIP is a method of creating highly polarized nuclear spins in solution utilizing the spin order of para-hydrogen^18^. The polarization transfer occurs following a hydrogenation reaction of an unsaturated molecular precursor with the para-hydrogen molecule. A variant of the PHIP approach uses a reversible interaction with the para-hydrogen molecule and is termed signal amplification by reversible exchange (SABRE)^19^. Both techniques offer orders of magnitude increase in the NMR signal for multinuclei. To date, we have seen a couple of reports of hyperpolarized ^31^P in solution—done by the SABRE method^20, 21^. However, the ^31^P hyperpolarization achieved in these studies was of molecules that are not water soluble and do not present any biomedical relevance.

Phosphate-containing compounds are of key importance in biology and medicine and the 100% natural abundance of their NMR-active ^31^P nucleus make them affordable, attractive targets. Here, we show that observation of hyperpolarized ^31^P in important phosphate-containing molecules is indeed possible with the d-DNP methodology under physiologically relevant conditions. We present a potential application of this capability for fast pH sensing. We note that there are numerous important phosphate-containing metabolites in biology and medicine. Further research into their metabolism and bodily distribution is likely to benefit from the approach described herein.

## Results

### Inorganic phosphate (Pi) is observed in a hyperpolarized state in aqueous media

To explore the hyperpolarized state of Pi in solution, the following formulation was prepared: 120.1 µmol KH_2_PO_4_, 15.1 µmol OXO63 radical, 5.4 mmol glycerol, and 33.1 mmol H_2_O. A vitrification assay^22^ showed that this formulation indeed formed a glass upon rapid freezing to cryogenic temperature (liquid nitrogen). About 100–120 mg of this formulation were placed in a sample cup and polarized for 1.5–3 h with a microwave frequency of 94.097 GHz, a power of 100 mW, and a temperature of 1.40–1.46 K at a 3.35 T magnetic field using a d-DNP setup. Then the formulation was quickly dissolved in 4 ml of aqueous media at a temperature of 170 °C and a pressure of 10 bar. The dissolved hyperpolarized solution was directly injected to a 10-mm NMR tube in a 5.8 T NMR spectrometer and ^31^P spectra were continuously recorded. The hyperpolarized signals were recorded starting about 16 s from the start of the dissolution process.

**Fig. 1:**
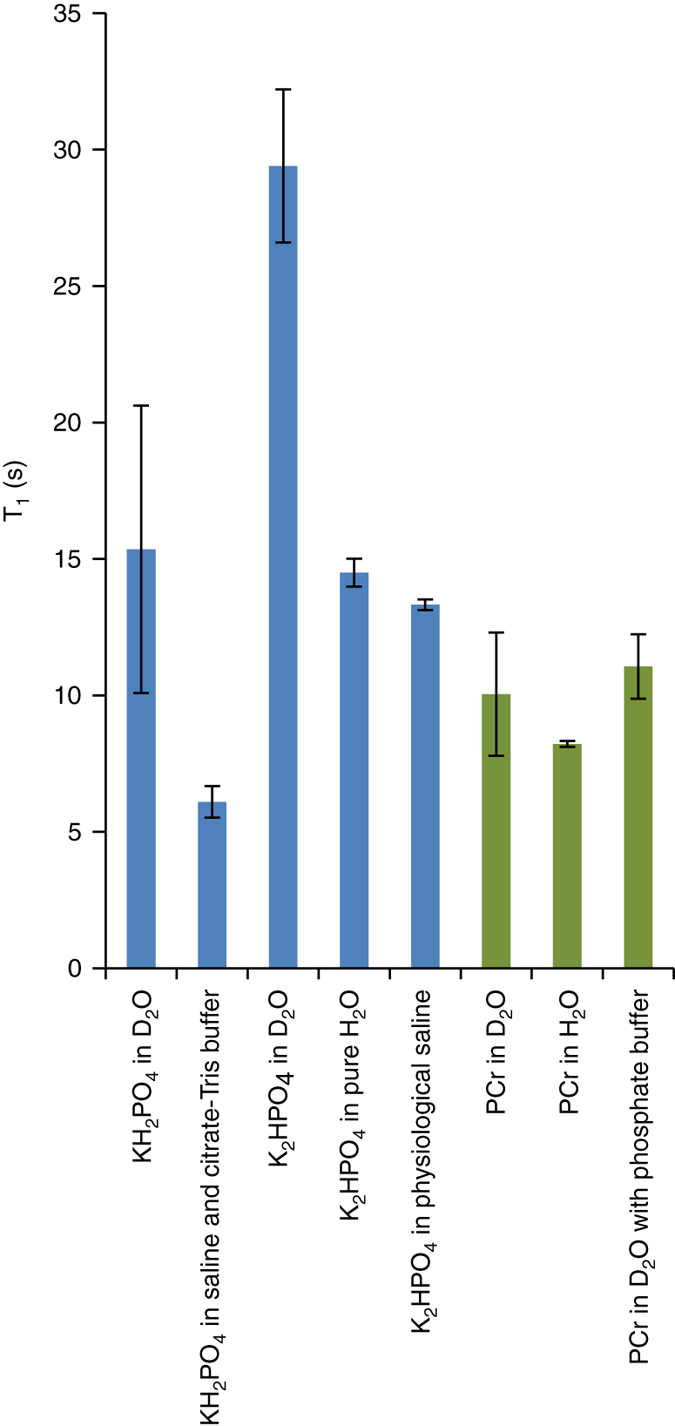
Summary of T_1_ relaxation time constants for hyperpolarized phosphates in aqueous media. The average value of *n* measurements is given and the error bars represent the standard deviation. The hyperpolarized decay curves were recorded at room temperature. The concertation of the hyperpolarized phosphate, the pH, and the number of measurements for each condition was as follows: KH_2_PO_4_ in D_2_O: 0.94 ± 0.14 mM, pH 5.1, *n* = 2; KH_2_PO_4_ in saline and citrate-Tris buffer: 0.43 ± 0.07 mM, pH 4.5, *n* = 2, includes two experiments with N_2_ bubbling and two experiments without N_2_ bubbling. K_2_HPO_4_ in D_2_O: 1.80 ± 0.90 mM, pH 8.1, *n* = 5 K_2_HPO_4_ in pure H_2_O: 2.95 ± 0.03 mM (*n* = 3), 34.93 mM (*n* = 1), pH 8.1 (altogether four experiments; one experiment was performed with a 2 M concentrated formulation of Pi, resulting in a high concentration in the NMR tube). K_2_HPO_4_ in physiological saline: 1.14 ± 0.02 mM, pH 8.1, *n* = 2 PCr in D_2_O: 2.17 ± 0.71 mM, pH 6.5, *n* = 2; PCr in H_2_O: 2.78 ± 0.00 mM, pH 7.4, *n* = 2; PCr in D_2_O with phosphate buffer: 1.20 ± 0.17 mM, pH 7.4, *n* = 2. *Pi* inorganic phosphate, *PCr* phosphocreatine

When dissolved in D_2_O, the Pi signal was clearly visible for about 50 s across 50 scans, each acquired with a 10° nutation angle. The T_1_ of this hyperpolarized site was about 15 s (Fig. 1) and the pH of the hyperpolarized media was 5.1. To explore the effect of a protonated solvent and the addition of osmotic pressure at physiological levels, the same experiment was repeated with water instead of D_2_O. The dissolution medium consisted of 90% physiological saline and 10% D_2_O with 100 mg/l EDTA, which were combined in the NMR tube with 5 ml of citrate-Tris buffer (altogether 300 mOsm and a pH of 4.5). Both the dissolution solvent and the citrate-Tris buffer were bubbled with N_2_ for 1 h prior to use. The hyperpolarized Pi signal was again clearly visible but the T_1_ appeared to be shortened to about 6 s (Fig. 1). This result could be expected as T_1_ shortening by protonated solvents and by the addition of osmotic pressure to the solution was previously seen for other hyperpolarized compounds^23, 24^. To check for possible effects of oxygen depletion, the experiment was repeated twice more in fresh solutions without N_2_ bubbling. The resulting T_1_ of the hyperpolarized Pi was similar at 6 s. Therefore, the results with and without N_2_ bubbling were combined and are presented in Fig. 1.

### A basic environment prolonged the T_1_ of hyperpolarized Pi

**Fig. 2:**
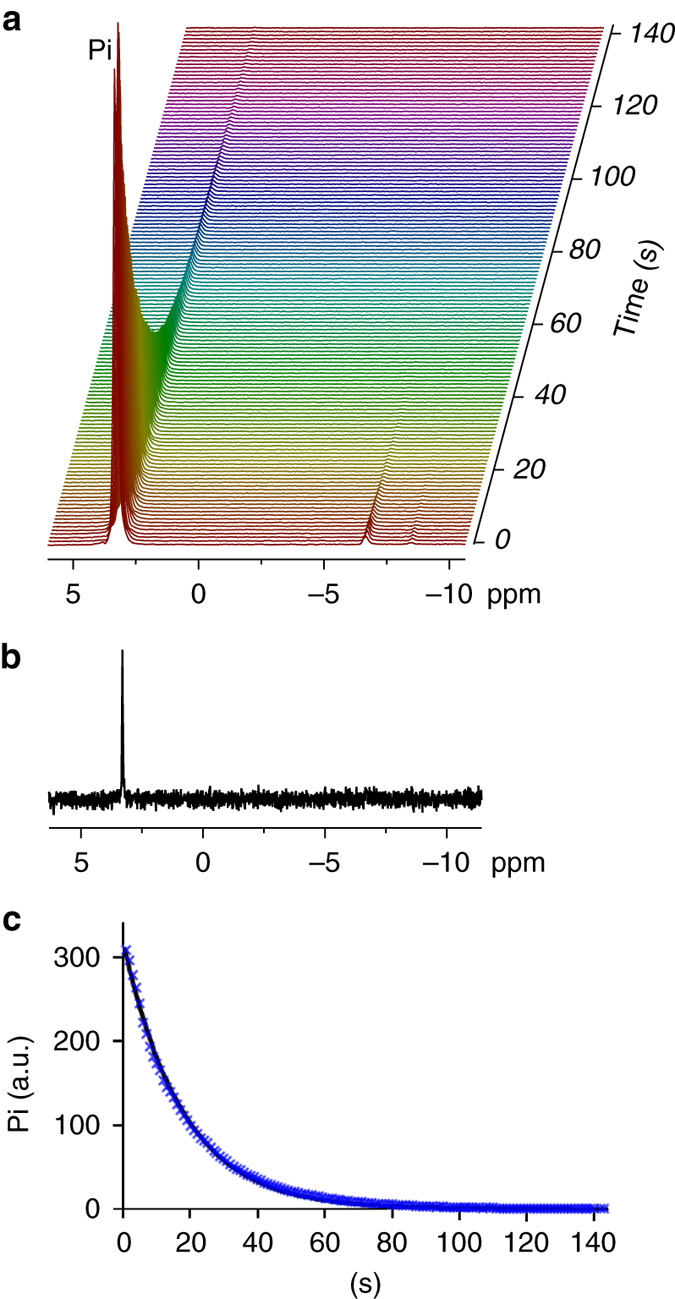
High-resolution ^31^P NMR spectra of Pi in a hyperpolarized state and at thermal equilibrium. **a** ^31^P NMR spectra of hyperpolarized Pi in D_2_O (2.72 mM). The solid-state polarization was carried out in basic pH and the dissolution medium pH was 8.1 (with the Pi). The individual spectra, acquired with 1 s temporal resolution, are presented using a Maroon color scale for visual clarity and convenience. A total of 180 scans were acquired. Only the spectra with visible Pi signal are shown. The excitation angle was 10° at a field of 5.8 T. The initial polarization demonstrated in the 1st spectrum was 9.6%. The line width of the hyperpolarized signals was 13 Hz. **b** A thermal equilibrium ^31^P spectrum of the same sample shown in **a**, acquired with 400 excitations, a nutation angle of 90°, and a repetition time of 151 s (*ca*. 17 h of acquisition). **c** The decay of the hyperpolarized Pi signal is demonstrated via the integrated intensity of the Pi signal (in arbitrary units, a.u.), marked with blue x. The fit of the data to the longitudinal relaxation decay equation (see “Methods”) is depicted by the continuous line (*black*). This fit resulted in a T_1_ of 24.2 s (95% confidence interval 23.7, 24.6 s)

We further explored the effect of pH of the solid-state formulation (and the resulting hyperpolarized media) on the production and maintenance of the hyperpolarized state. To this end we used K_2_HPO_4_ to prepare the formulation for polarization instead of KH_2_PO_4_, keeping all other conditions the same. When dissolved in D_2_O, this formulation resulted in a pH of 8.1. This change led to an improvement in the ability to observe the hyperpolarized state of Pi, as the T_1_ in solution increased to about 29 s (Fig. 1). Figure 2 demonstrates a typical experiment done at these conditions. The hyperpolarized signal of Pi in D_2_O is clearly visible for more than 2 min across more than 120 acquisitions, each acquired with a 10° nutation angle. The thermal equilibrium spectrum of this sample is shown in Fig. 2b. The enhancement factor for the hyperpolarized Pi signal at these conditions was calculated to be 11,364 ± 438 (at 5.8 T, *n* = 2, 1.5 h of polarization), equivalent to 9.3 ± 0.3% polarization. In addition, we observed two other hyperpolarized signals downfield from Pi, likely corresponding to contaminations of the K_2_HPO_4_ batch.

### The effect of water protons on the hyperpolarized Pi decay

To explore the effects of solvent (water) protonation in the basic condition, the experiment was repeated using pure water for dissolution. The T_1_ of the hyperpolarized Pi decreased twofold to about 15 s (Fig. 1). This effect can be explained by inter-molecular dipolar interaction between the water protons and the hyperpolarized ^31^P nucleus. The effect of dissolution osmolarity was also tested (using physiological saline as the dissolution medium, *ca*. 300 mOsm), and the T_1_ was found to be shortened slightly to about 13 s (Fig. 1). Despite this T_1_ shortening effect of using water instead of D_2_O and using iso-osmotic solutions, the T_1_ of the basic formulation was still comparable to the T_1_ of the acidic formulation in D_2_O. We conclude that the basic formulation is preferential for preparing Pi solutions for hyperpolarization.

### Media components affect the Pi T_1_ relaxation rate too

Thus far we investigated the T_1_ of Pi under varied solution conditions in a hyperpolarized state. These conditions included protonation of the solvent, mixture pH, and osmotic pressure. However, the T_1_ of Pi could be sensitive to other medium components as well. To explore this aspect further we performed a preliminary investigation comparing two different types of media (water and citrate-Tris media). This investigation was carried out at thermal equilibrium and is summarized in Supplementary Note 3 and Supplementary Figs. 3 and 4. Briefly, we have found a surprising T_1_ prolongation of the citrate-Tris media on the T_1_ of Pi at the acidic to neutral pH values (1 ≤ pH and lower than 7). The T_1_ values determined at thermal equilibrium (in both types of media) are in agreement with the T_1_ values determined in the hyperpolarized state (which are summarized in Fig. 1).

### Hyperpolarized Pi reports on instantaneous pH changes

**Fig. 3:**
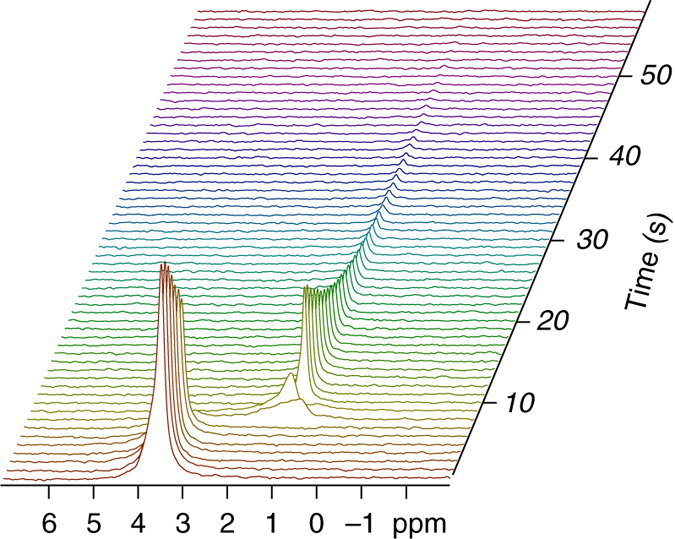
^31^P NMR spectra of hyperpolarized Pi at two different pH values. These spectra demonstrate the ability for fast monitoring of pH changes with hyperpolarized Pi. The repetition time in this experiment was 1 s and the nutation angle was 10°. The initial pH was determined in a separate measurement to be 8.1. At about 9 s, 0.5 ml of citrate-Tris buffer in pH of 4.5 was mixed into the solution. The final pH was determined in the same sample to be 4.8. The wide signals in spectra 8 and 9 are due to the mixing process, which involved air bubbles going through the sample during the measurement. The line widths of the hyperpolarized signals were 9 and 7 Hz at pH 8.1 and 4.8, respectively

The chemical shift of Pi is known to be sensitive to pH at the most physiologically relevant pH ranges^25^ (Supplementary Note 2 and Supplementary Fig. 1). To illuminate an aspect of biomedical significance, we decided to demonstrate that hyperpolarized Pi can report on instantaneous pH changes. To this end we carried out a couple of experiments in which the hyperpolarized Pi signal underwent through varying and controlled pH regimes within one hyperpolarization decay time and was recorded continuously. The first experiment is described below and the second is shown in Supplementary Note 4 and Supplementary Fig. 5. In the experiment depicted in Fig. 3, a basic formulation of Pi was hyperpolarized as described in the “Methods”. The hyperpolarized sample was dissolved in 4 ml of medical grade saline solution with 10% D_2_O and immediately transferred from the d-DNP device to the NMR spectrometer. The pH of this solution composition was determined independently prior to the hyperpolarized experiment to be 8.1. About 9 s after the appearance of the hyperpolarized signal, the pH in the sample tube was quickly acidified by means of a quick injection of 0.5 ml of citrate-Tris buffer of pH 4.5. In response, the continuously recorded ^31^P spectra show two spectra with broad lines attributed to air bubbles introduced during mixing and then the following spectra show the hyperpolarized ^31^P signal at a new chemical shift representing the new pH of 4.8, determined in the same sample immediately after the end of the hyperpolarized decay. This result demonstrates the potential for instantaneous pH sensing of aqueous solutions using hyperpolarized Pi. Since Pi is the ultimate pH sensor for biology, this result brings hope that hyperpolarized Pi can be used for clinical imaging of pH.

### ^31^P hyperpolarization of phosphocreatine (PCr) is feasible

**Fig. 4:**
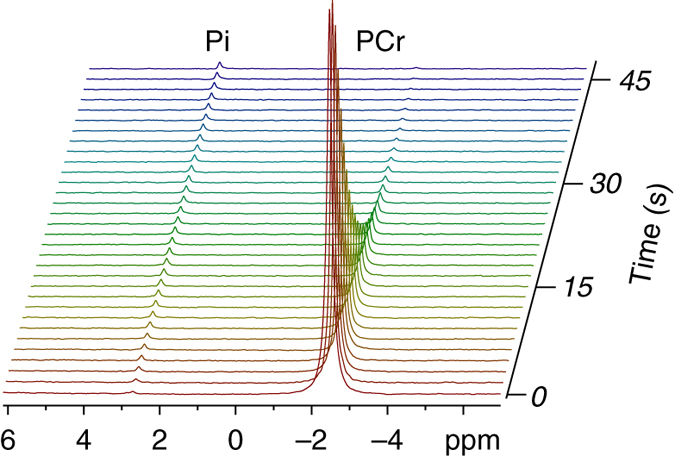
^31^P spectra of hyperpolarized PCr in D_2_O containing 50 mM phosphate buffer (pH 7.4). The Pi signal is shown to increase over time and then plateau. This is due to the magnetization of the Pi ^31^P nuclei from the dissolution media (not hyperpolarized), reaching steady state at thermal equilibrium in the magnetic field of the spectrometer. The hyperpolarized PCr signal decays with time to thermal equilibrium. The thermal equilibrium signal of PCr is undetectable in one scan due to its low concentration (2.78 mM). The chemical shift was assigned using the PCr signal at −2.5 ppm^34^. Each spectrum was acquired with a nutation angle of 15° and the repetition time was 1.5 s

PCr is a phosphate-containing molecule of key biochemical importance. Its main biochemical activity relates to the creatine kinase (CK) reaction, which serves to regenerate ATP in cells that rapidly consume ATP such as brain and muscle cells. To test the ability to observe the PCr phosphate in a hyperpolarized state, the following formulation was prepared: 25.4 μmol PCr, 1.8 μmol OXO63, 0.9 mmol glycerol, 3.6 mmol D_2_O. About 40–80 mg of this formulation were polarized in the same conditions described above. Indeed, we were able to detect the hyperpolarized state of the ^31^P nucleus in the PCr molecule in aqueous media as well. Figure 4 demonstrates the hyperpolarized signal of PCr, visible for about 40 s, when dissolved in D_2_O containing 50 mM phosphate buffer and 2 mg of the CK protein (not active at the time of measurement), at a final pH of 7.4. The T_1_ of this hyperpolarized site was about 11 s (Fig. 1). The CK protein was added to the NMR tube to test the ability to observe not only the PCr phosphate in a hyperpolarized state but also in the presence of the enzyme that may bind it and therefore may affect the decay of the hyperpolarized state.

The effects of solvent protonation and osmolarity were investigated for PCr as well and the results are summarized in Fig. 1. The T_1_ of PCr did not appear to be significantly affected either by the water protonation or the osmolarity of the media. An important property of the PCr phosphate is that its ^31^P chemical shift is much less sensitive to pH changes in the physiological range, because its pKa is below the physiological range, at pH 4.3^25^.

## Discussion

Magnetic resonance (MR) imaging provides the most exquisite anatomical soft tissue details and therefore contrast in medical imaging that relies on MR—and can be fused to conventional magnetic resonance imaging (MRI) images—is preferential. Hyperpolarized compounds have the potential to serve as molecular imaging probes on clinical imaging and to provide unique contrast mechanisms that will report on tissue functionality without ionizing radiation. Here, we capitalized on the d-DNP technique to create an unexplored type of molecular imaging probes for MR, i.e., hyperpolarized phosphates. We demonstrated the ability to hyperpolarize the ^31^P nucleus in Pi and PCr, which warrants research into many more endogenous phosphates.

We note that a key factor in the success of hyperpolarization of phosphates for use in solutions is a relatively long T_1_ in solution. This requirement applies to any spin hyperpolarized MR molecular imaging probe. Indeed, Pi and PCr have a relatively long T_1_ in aqueous solutions at 5.8 T compared to other phosphates; although in the d-DNP field these are considered among the shortest T_1_ that have been amenable for investigation. A detailed investigation of ^31^P T_1_ in aqueous media is provided in Supplementary Notes 1–3, Supplementary Table 1, and Supplementary Figs. 2–4. Therefore, as a first step in the development of further phosphate compounds for hyperpolarization it is important to characterize their T_1_ time constants.

Another key factor for the utilization of hyperpolarized phosphates is the actual percent polarization achievable in solution. This polarization level is intimately dependent on the solution state T_1_ of the particular nucleus in the specific molecule, solvent, mixture, temperature, pressure, and magnetic field throughout the path of the hyperpolarized media to the spectrometer and within it. For d-DNP studies, the polarization level in solution is primarily dependent on the polarization level achieved in the solid state, which is dependent on the formulation, microwave irradiation frequency, temperature, magnetic field, and the duration of polarization. Nevertheless, the following is a brief review of previously reported polarization levels in solution. We have demonstrated here ^31^P polarization levels of 9.3%. This polarization level is within the same order of magnitude of ^13^C polarization achieved on d-DNP studies (e.g., 5.5%^26^, 4.2%^23^, and 24%^27^) and is higher than the ^31^P polarization level achieved using para-hydrogen-based experiments (2.3%^20^, 0.3%^21^). Other examples of polarization levels previously documented in solutions are 1.3–9.1% for ^19^F^15^, and 0.009^10^ to 2%^9^ for ^1^H.

To demonstrate one potential biochemical application of hyperpolarized Pi, we carried out experiments in which the hyperpolarized Pi reported on the pH of the microenvironment in a rapid pH change process, with a temporal resolution of 1 s. pH sensing in intact tissues in living subjects is a long sought target with immediate diagnostic and therapeutic implications in various diseases and conditions^25^. Pi has long been recognized as a promising probe for imaging tissue pH^28, 29^, as (1) its chemical shift is sensitive to changes of pH in the range of 6.4–7.6, which is the relevant physiological range (Supplementary Note 2 and Supplementary Fig. 1), (2) it is a non-toxic endogenous substrate present in high concentrations physiologically (0.3–1.1 mM in blood^30^), (3) it requires no expensive isotopic labeling as ^31^P is 100% naturally abundant, and (4) its gyromagnetic ratio is high (γ_31P_/γ_1H_ = 0.40). However, the clinical relevance of Pi for pH sensing has been limited by two factors. First, the signal-to-noise ratio (SNR) of in vivo ^31^P spectra is low due to the relatively long T_1_ relaxation time of Pi combined with the inherently low SNR ratio of thermal-equilibrium ^31^P MR, dictating acquisition times that are too long to be clinically useful. Second, previous studies have shown that the endogenous Pi signal appearing on in vivo ^31^P spectra predominantly originates from the intracellular space^29^, which is not a sensitive indicator of disease, unlike extracellular pH that has been shown to vary in a large number of important diseases and to be a marker of tissue changes in response to ischemia^31^, malignancy^32^, and metastasis^29^. The approach described herein surmounts these two obstacles: (1) the d-DNP method provides a very high SNR and thus allows detection of lower quantities (which can be translated to higher spatial and temporal resolution on imaging); (2) unlike endogenous Pi, the hyperpolarized Pi developed here could serve as a potential reporter of extracellular pH, as, depending on transport kinetics, it will likely be primarily located in the extracellular space during the short T_1_ limited window of observation.

The following rough calculation is provided to give an estimate for the potential utility of Pi in vivo, considering the signal enhancement demonstrated herein. The concentration of Pi in the hyperpolarized media measured here was *ca*. 1 mM. We shall consider the case in which such a concentration is reasonably achieved in the tissue. The detected volume in our probe was about 1.3 ml. Let us consider a potential spatial resolution of 5 × 5 mm^2^ and a slices thickness of 5 mm. This results in voxels with a volume of 125 mm^3^. Thus, in the volume detected in the current experiment (1.3 ml), there are about 10 such potential voxels. All of the hyperpolarized studies presented here show an SNR of more than 100 (per 1 mM), even after ca. 15–20 s that would be required for circulation through the vasculature. Therefore, it appears that an in vivo observation is warranted with a potential SNR higher than 10 (per 1 mM per 5 × 5 × 5 mm^3^ voxels). Many more factors should be taken into account: one factor that will increase the SNR on imaging is the fact that one actually excites each voxel many times, depending on the acquisition sequence, thereby increasing the SNR of each voxel correspondingly. Factors that may reduce the potential signal are (1) a reduced sensitivity of the in vivo radio frequency (RF) coils in a pre-clinical MRI compared to the high resolution NMR spectrometer and 10-mm probe used here, and (2) possible T_1_ shortening by blood and tissues. The sensitivity of RF coils can be optimized and should be checked back-to-back with the relevant coils. The main shortening effect in blood and tissues is likely due to oxygen. In our hands, oxygen did not dramatically shorten the T_1_ of hyperpolarized Pi (Supplementary Note 5). In short, in vivo experimentation is warranted keeping in mind that each part of the detection system (sequences and hardware) should be optimized for the detection of hyperpolarized ^31^P nuclei.

We note that NMR chemical shifts are relative properties and therefore a chemical shift standard would be required for hyperpolarized Pi to actually serve as such a reporter on tissue pH. However, the SNR of the hyperpolarized Pi is much larger than the thermal equilibrium SNR meaning that other endogenous phosphates, which could potentially serve as references for chemical shift, are unlikely to be observed simultaneously with the hyperpolarized Pi. One potential solution to this problem would be to generate a map of relative differences of all of the observed chemical shifts. Another potential solution would be to combine another hyperpolarized phosphate with a chemical shift that is insensitive to pH. Hyperpolarized PCr which was developed here is a potential candidate for this purpose as its chemical shift is insensitive to pH at the physiological pH range. Thus, by combining two agents with different chemical shifts and different chemical shift pH sensitivities, one could accurately determine the pH of the microenvironment.

We note that using the d-DNP technique^1^, it was previously shown that detection of hyperpolarized bicarbonate and carbon dioxide provides a measure of tissue pH^12, 33^. However, the particular tissue compartment inspected in this way is inherently dependent on the hyperpolarized substrate administered to the subject (pyruvic acid^33^ or bicarbonate^12^) and the kinetics of membrane transport and metabolism in the specific tissue inspected^31^.

Here, we propose that hyperpolarized Pi could serve as a robust pH sensor for tissue pH. The data suggest that hyperpolarized Pi could be imaged with high SNR on a time scale of seconds (calculation above). In addition, co-polarization and dissolution of Pi with PCr can provide an accurate internal standard for chemical shift.

The solid-state polarization, and therefore the SNR of hyperpolarized phosphates in solution, could likely be improved by further formulation trials. However, we note that in the current state of the art of d-DNP instruments, it is not possible to monitor the solid-state build-up of ^31^P polarization. This makes formulation development for ^31^P DNP cumbersome and costly as the polarization build-up can only be monitored after dissolution of the hyperpolarized sample. Further studies are required to robustly characterize the solid-state build-up time constants of Pi and PCr and the factors that may affect them. Such studies are currently underway in our laboratory.

In summary, we have observed here a DNP-driven hyperpolarized state of ^31^P in aqueous solutions. Using this technology, this state was observed in two biochemically important molecules, Pi and PCr. Factors such as solvent de-protonation, a basic pH, and media composition increased the T_1_ of Pi and therefore also increased the time duration at which this signal could be observed. While the comparably short T_1_ remains a challenge, this work warrants further research in other important phosphate-containing biological molecules that could potentially shed light on fundamental biochemical and physiological processes.

## Methods

### Chemicals

K_2_HPO_4_, KH_2_PO_4_, and PCr (as disodium salt) were purchased from Sigma-Aldrich, Rehovot, Israel. The OXO63 radical (GE Healthcare, UK) was obtained from Oxford Instruments Molecular Biotools (Oxford, UK). The citrate-Tris medium used here contained 100 mM citrate, 10 mM KCl, and 95 mM TRIS, all purchased from Sigma-Aldrich.

### DNP spin polarization and dissolution

Spin polarization and fast dissolution were carried out in a d-DNP set-up (HyperSense, Oxford Instruments Molecular Biotools, Oxford, UK). A microwave frequency of 94.097 GHz was chosen based on the work of Reynolds et al^16^. In this report, a solid-state polarization of ^31^P was achieved when the microwave irradiation frequency was set at 0.017 GHz below the microwave frequency required to achieve solid-state polarization of the ^13^C nucleus of pyruvic acid. In our spin polarizer, the latter frequency corresponds to 94.114 GHz.

The dissolution process was performed as previously described^1^ and detailed in the results section. Briefly, 40–120 mg of the ^31^P containing formulation which were placed in a the polarization sample cup were quickly dissolved in 4 ml of superheated aqueous media (170 °C and 10 bar). The dissolved hyperpolarized solution was directly injected to a screw cup 10-mm NMR tube in a 5.8 T NMR spectrometer via a Polytetrafluoroethylene (PTFE) line of about 2.4-m length with 6 s of He (g) chase. ^31^P spectra were continuously recorded immediately at the start of the dissolution process. The hyperpolarized signals appeared in the spectra at about 15–16 s from the start of the dissolution process (meaning that the dissolution process and the chase of the media into the NMR tube occurred within about 15–16 s).

### ^31^P NMR

^31^P NMR spectroscopy was performed in a 5.8 T NMR spectrometer (RS2D, Mundolsheim, France), using a 10-mm broad band NMR probe. Unless otherwise stated, the chemical shift scale of the spectra presented herein was calibrated based on a separate measurement of an ATP standard sample (127 mM in D_2_O, pH 7), carried out prior to each ^31^P acquisition, calibrating the α-ATP signal to −10.03 ppm^34^.

### Processing and data analysis

Spectral processing was performed using MNova (Mestrelab Research, Santiago de Compostela, Spain). Integrated intensities were calculated either with MNova or with DMFIT^35^.

Determination of the T_1_ of the hyperpolarized sites was performed by curve fitting of the signal decay to the following equation: $M\left( t \right) = {M_o}*{{\rm{e}}^{\left( {\frac{{ - t}}{{{T_1}}}} \right)}}*\cos {\theta ^{\left( {\frac{t}{{TR}}} \right)}}$, in which TR, the time between excitations, and *θ*, the nutation angle of excitation, are known.

Curve fitting was performed using Matlab (Mathworks, Natick, MA, USA).

The absolute enhancement factor was determined by comparing the maximal integrated signal intensity obtained under hyperpolarized conditions to the integrated signal intensity of the same sample at thermal equilibrium acquired with the same nutation angle under fully relaxed conditions. In order to compare the integrated intensities from the thermal and hyperpolarized acquisitions the same spectral acquisition parameters (spectral width, number of points, receiver gain) and processing (apodization, zero-filling) were used and the thermal equilibrium was corrected for number of scans.

### Data availability

The data that support the findings of this study are available from the corresponding author on request.

## Electronic supplementary material

Supplementary Information
