# Author Correction: Biochemical phosphates observed using hyperpolarized ^31^P in physiological aqueous solutions

**DOI:** 10.1038/s41467-018-04538-5

**Published:** 2018-05-22

**Authors:** Atara Nardi-Schreiber, Ayelet Gamliel, Talia Harris, Gal Sapir, Jacob Sosna, J. Moshe Gomori, Rachel Katz-Brull

**Affiliations:** Department of Radiology, Hadassah-Hebrew University Medical Center, Jerusalem, Israel

Correction to: *Nature Communications* 10.1038/s41467-017-00364-3, published online 24 August 2017

The original version of the Supplementary Information associated with this Article contained an error in Supplementary Figure 2 and Supplementary Figure 5 in which the ^31^P NMR spectral lines were missing. The HTML has been updated to include a corrected version of the Supplementary Information.

