# Supplementary material for: Biochemical phosphates observed using hyperpolarized 31P in physiological aqueous solutions
Source: Nat Commun. 2017 Aug 24;8:341. doi: 10.1038/s41467-017-00364-3 (PMC5570947; doi:10.1038/s41467-017-00364-3)
Supplement: Supplementary file 1 — Supplementary Information [file 41467_2017_364_MOESM1_ESM.pdf]

Supplementary Note 1: Determination of ^{31}P T_1 of Pi, PCr, and ATP at thermal equilibrium.

The ^{31}P nuclei of P_i and PCr have been previously investigated and were shown to have T_1s of 2 - 5 s in biological samples [1-4]. While the T_1 of PCr showed a strong field dependence [1, 5] the signal of Pi is not expected to behave in the same way. The reason for this is that the contribution of chemical shift anisotropy to the longitudinal relaxation of this molecule is very low due to its symmetric and tetrahedral structure [6]. Our ability to visualize the ^{31}P nuclei in these molecules in a hyperpolarized state as opposed to larger phosphate containing molecules such as ATP is likely related to the relatively long T_1 of these sites in solution. Because these T_1s had not been determined previously at 5.8 T (to the best of our knowledge) we have determined their value here, at thermal equilibrium, in the same spectrometer used for the current hyperpolarized studies. This investigation was carried out in hyper-osmotic aqueous solutions, in two pH values, and therefore does not cover the full range of solution compositions for which T_1s were determined in the course of the hyperpolarized studies. The results are summarized in the Supplementary Table 1 and highlight the difference between the T_1s of Pi and PCr and those of the ATP phosphates. The full range of pH is described in Supplementary Figure 2 (in low to iso-osmotic solutions).

Supplementary Table 1: Longitudinal relaxation times* of Pi, PCr, and ATP

Sample composition and pH	^{31}P containing compound	T_1 (s)	n
ATP 41 mM<br>PCr 42 mM<br>KH_2PO_4 103 mM<br>NaCl 108 mM<br><br>pH 7.4	Pi	6.8 ± 0.2	2
	PCr	6.1 ± 0.5	2
	α-ATP	1.2 ± 0.2	2
	β-ATP	1.2 ± 0.1	2
	γ-ATP	1.2 ± 0.1	2
ATP 56 mM<br>KH_2PO_4 143 mM<br>NaCl 150 mM<br><br>pH 5	Pi	6.6 ± 0.2	4
	α-ATP	1.2 (0.8 , 1.6)	1
	β-ATP	0.9 (0.5 , 1.2)	1
	γ-ATP	1.2 (1 , 1.5)	1

* All measurements were carried out at thermal equilibrium in hyper-osmotic aqueous solutions at 5.8 T. T_1 was determined using the steady state variable nutation angle (SSVN) method [7] at room temperature. n, number of experiments. Results are given as mean ± standard deviation for n>1, or with 95% confidence intervals of curve fit for n=1.
ATP and PCr were purchased as disodium salts (Sigma-Aldrich, Rehovot, Israel).

Supplementary Note 2: The dependence of Pi chemical shift on pH.

The Pi molecule is considered the perfect pH sensor because its chemical shift changes significantly at the physiological relevant pH range. Supplementary Figure 1 shows the theoretical dependence of the ^{31}P chemical shift of Pi on pH at this range. The graphs were plotted using the Henderson–Hasselbalch equation using the pKa and the acidic and basic chemical shift values reported by Soto *et al.* [8].

Supplementary Figure 1: Theoretical chemical shift of Pi dependence on pH.

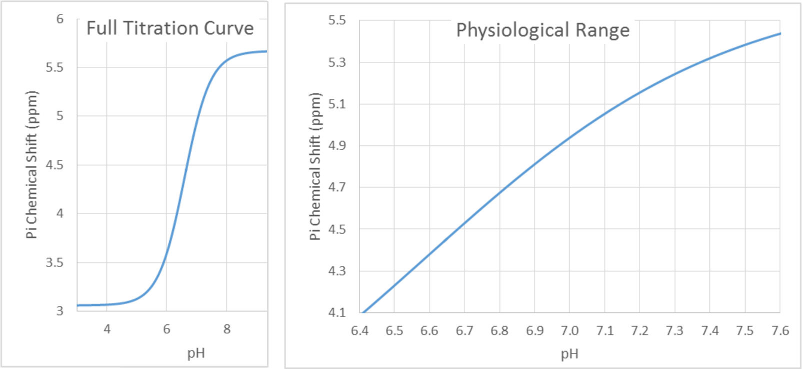

Since the Pi molecule contains 3 protonation sites and has 3 pKa values (pKa_1 = 2.12, pKa_2 = 7.21, and pKa_3 = 12.67 at 25 °C [9]), the chemical shift dependence of Pi on pH in the full pH range is more complex. In Supplementary Figure 2 we show the Pi chemical shift determined here experimentally across the pH range of 1 – 13.5. The Pi samples in varied pHs were determined in two types of media: 1) Saline - 150 mM sodium chloride in 10:90 D_2O:H_2O, and 2) citrate medium - citrate-TRIS medium (composition provided in Methods, main text). The media were titrated with HCl or NaOH to achieve the desired pH per sample.

Supplementary Figure 2: Experimental chemical shift of Pi - dependence on pH.

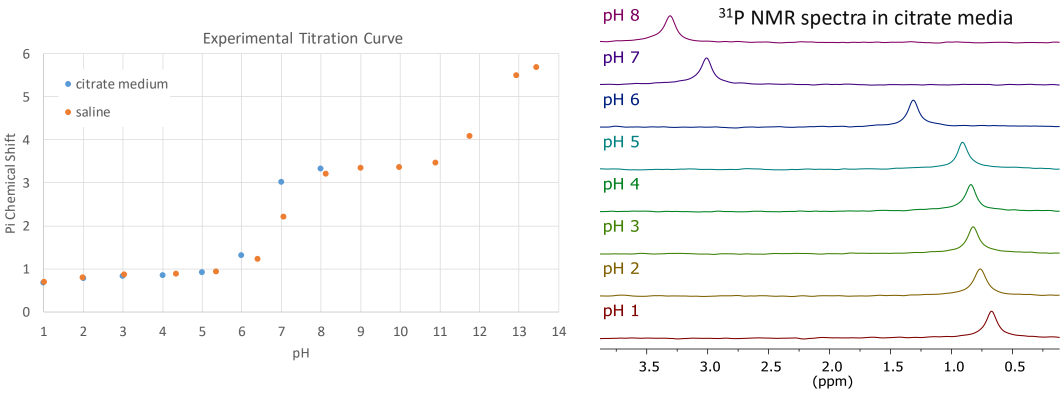

Supplementary Note 3: T_1 values of Pi at various pH values in different solutions.

Solution type 1 – water

Supplementary Figure 3 summarizes the ^{31}P T_1 of Pi in 50:50 D_2O:H_2O. The same Pi solution sample (starting concentration 33.3 mM, starting volume 3 ml) was titrated with HCl or NaOH to reach the desired pH values. The solution contained also PCr at the same concertation. Osmolarity increase was not recorded. T_1 at the various pH values was determined with the steady-state variable nutation angle method (SSVN) [7]. The SSVN measurement was repeated 2-4 times per sample with varying repetition times (TR) to ensure reproducibility and accuracy. The green arrows mark the approximate pKa values based on previously published data [9]. We note that the pKa is dependent on the mixture composition and was not re-determined here. The chemical shift of the PCr signal was not altered in response to pH changes.

As can be seen, at the range of the basic pH values 8 ≤ pH ≥ 10 the T_1 of Pi is longer than 8 s. However, at the acidic-neutral pH values, 2 ≤ pH ≥ 7, the T_1 values are shorter ranging at about 1 - 3 s.

Changes in T_1 trends with pH can be seen next to the 3 pKa values of Pi, (marked by the green arrows). The results do not suggest a uniform trend that could be applied to all 3 pKa values. This result suggests that changes in pH in this medium type do not affect a single dominant longitudinal relaxation mechanism.

Supplementary Figure 3: T_1 of Pi in water – dependence on pH.

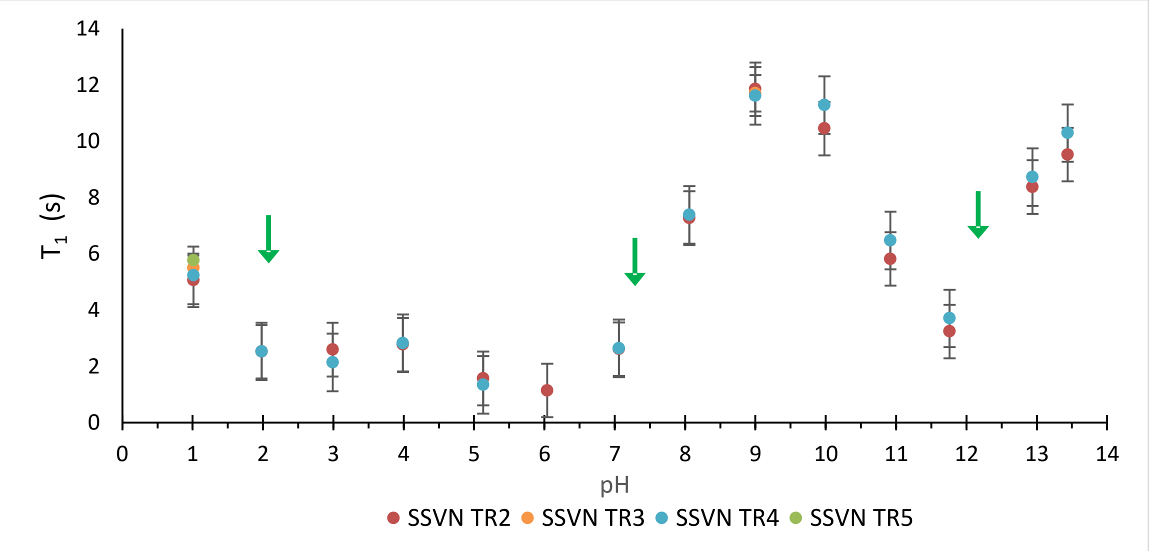

Solution type 2 – citrate-TRIS media

Supplementary Figure 4 summarizes the ^{31}P T_1 of Pi in 50 mM samples in citrate-TRIS medium containing 100 mM citrate, 10 mM KCl, and 95 mM TRIS. Eight such samples were prepared (3 ml) and each was titrated to the required pH with HCl or NaOH and transferred to an NMR tube. The volume increase due to titration was less than 5 %. Osmolarity increase was not recorded.

T_1 at the various pH values was determined with the SSVN method [7]. The SSVN measurement was repeated 2-4 times per sample with varying repetition times (TR) to ensure reproducibility and accuracy.

As can be seen, at this wide range of pH values (1 ≤ pH ≥ 8) the T_1 of Pi is not markedly changing and averages at 8.8 ± 1.2 s. Therefore, the dependence of the T_1 of Pi on pH is different in the citrate-TRIS media than in water. It appears that different relaxation mechanisms come into play in the citrate-TRIS medium compared to water. This result demonstrates the complex nature of Pi longitudinal relaxation. It appears that for hyperpolarized applications, the T_1 of Pi needs to be evaluated in the specific conditions to meet the minimal allowed T_1 for actual visualization of the hyperpolarized signal.

Supplementary Figure 4: T_1 of Pi in citrate-TRIS media.

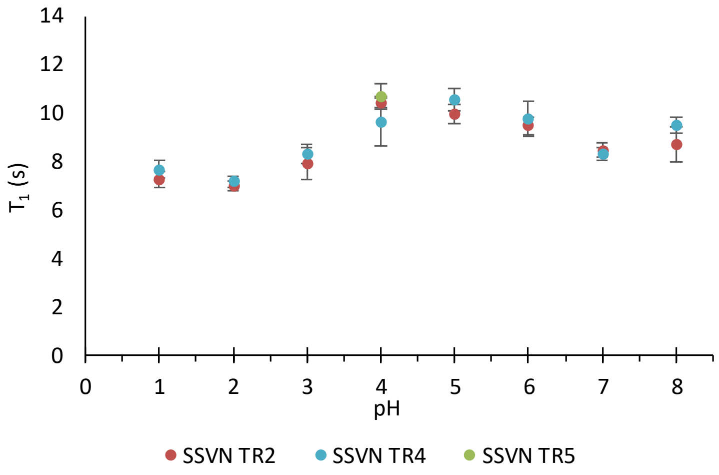

SSVN TR2, SSVN measurement with a repetition time of 2 s.
SSVN TR4, SSVN measurement with a repetition time of 4 s.
SSVN TR5, SSVN measurement with a repetition time of 5 s.

Supplementary Note 4: Real time pH dependent variation of hyperpolarized Pi chemical shift. In the main text we have shown an example of a pH change in the middle of recording a ^{31}P Pi hyperpolarized decay (Figure 3 in the main text). Supplementary Figure 5 shows another example of such an experiment where the chemical shift of the hyperpolarized Pi can be used to track pH changes in the solution. In this experiment, a basic formulation of Pi was hyperpolarized as described in the Methods. The hyperpolarized sample was dissolved in 4 ml of medical grade saline solution with 10% D_2O and immediately transferred from the d-DNP device to the NMR spectrometer. The pH of this solution composition was determined independently prior to the hyperpolarized experiment to be 8.1 (as in Figure 3 in the text). About 4 s after the appearance of the hyperpolarized signal, the pH in the sample tube was quickly acidified by means of a quick injection of 0.5 ml of citrate medium at pH 4.5 (citrate 100 mM, KCl 10 mM, titrated with TRIS to pH of 4.5). In response, the continuously recorded ^{31}P spectra show one spectrum with a broad line attributed to air bubbles introduced during mixing and then the following spectra show the hyperpolarized ^{31}P signal at a different chemical shift representing the new pH value of 4.8, determined independently on a similar composition prior to the hyperpolarized experiment (up to here this experiment is a reproduction of the experiment shown in Figure 3 in the main text). About 7 s after this first pH change another acidification step was introduced by means of a quick injection of 10 µl of concentrated HCl solution (diluted in 0.5 ml of H_2O to ensure arrival to the NMR tube) to the same sample. In response, the continuously recorded ^{31}P spectra show again one spectrum with a broad line attributed to air bubbles introduced during mixing and then the following spectra show the hyperpolarized ^{31}P signal at a different chemical shift representing the new pH value of 2.26, determined immediately after the end of the hyperpolarized decay in the same sample. Because in this second addition step the changes in chemical shift with pH are smaller, (as can be seen in Supplementary Figure 2), the hyperpolarized chemical shift change is less pronounced but is still clearly discernible. The 3 chemical shifts in which ^{31}P Pi resonated in this experiment are shown in the bottom panel for clarity. In the bottom panel the signals were plotted with the same intensity for visual clarity. Obviously the hyperpolarized signal decays with time and thus the noise in these three overlapping spectra appears increasing in this presentation form. This result further demonstrates the potential for instantaneous pH sensing of aqueous solutions using hyperpolarized Pi.

Supplementary Figure 5: ^{31}P NMR spectra of hyperpolarized Pi at three different pH values.

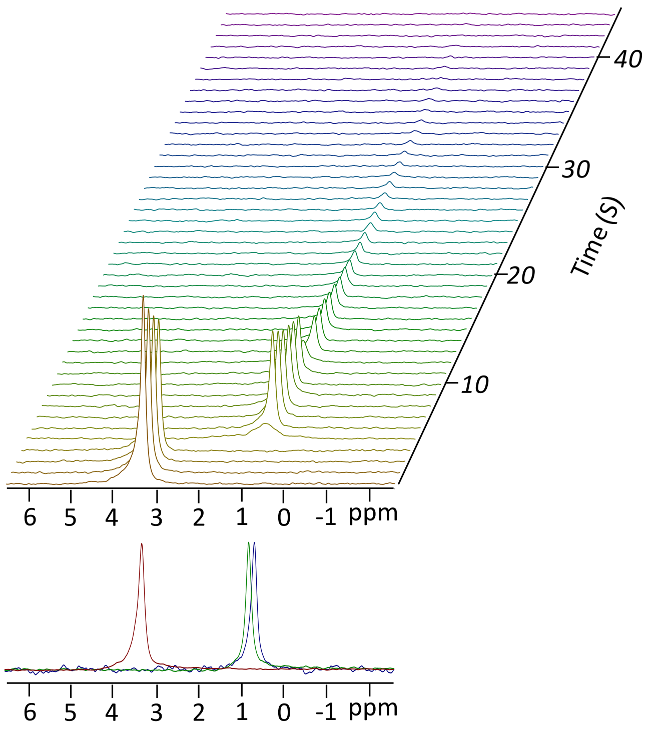

These spectra demonstrate the ability for fast monitoring of pH changes with hyperpolarized Pi. The repetition time in this experiment was 1 s and the nutation angle was 10 °. The initial pH was determined in a separate measurement to be 8.1. At about 4 s, 0.5 ml of citrate-TRIS buffer in pH of 4.5 was mixed into the solution. 7 s after the first injection, 0.01 ml of concentrated HCl diluted with 0.5 ml of H_2O was mixed into the solution. The final pH was determined in the same sample to be 2.26. The wide signals in spectra 5 and 12 are due to the mixing process which involved air bubbles going through the sample during the measurement. In the bottom panel signals of the three chemical shifts in which ^{31}P Pi resonated in this experiment were plotted with the same signal intensity for visual clarity.

Supplementary Note 5: Preliminary investigation of Pi T_1 in the presence of oxygen.

As a first step in evaluation of the feasibility of this phosphate group of hyperpolarized compounds we investigated the possible dependence of Pi T_1 on the presence of oxygen, the molecule which is responsible to much T_1 relaxation in blood.

To this end, the basic Pi formulation was polarized and dissolved in medical grade saline. ^{31}P spectra were continuously recorded with a repetition time of 1 s and a nutation angle of 10 ° (similarly to the experiment described in Supplementary Note 4). Fifteen seconds following the appearance of the first hyperpolarized signal the solution was mixed with 5 ml of medical grade saline saturated with oxygen by bubbling medical grade oxygen into the saline for 1 h prior to the combination with the hyperpolarized media. In this experiment, the initial T_1 of Pi (in the absence of oxygen saturation was 8.1 s. This T_1 relaxation time constant decreased to 5.7 s following the mixing with the media saturated with oxygen. This decrease in T_1 is expected, but is not dramatic and therefore warrants further experimentation with hyperpolarized Pi in biological systems.

Supplementary References

1. Lu, M., Chen, W. & Zhu, X.H. Field dependence study of in vivo brain P-31 MRS up to 16.4 T. *NMR Biomed.* **27**, 1135-1141 (2014).
2. Bottomley, P.A., Hardy, C.J. & Weiss, R.G. Correcting human heart P-31 NMR-spectra for partial saturation - evidence that saturation factors for PCr ATP are homogenuous in normal and disease states. *J. Magnet. Reson.* **95**, 341-355 (1991).
3. Matthews, P.M., Bland, J.L., Gadian, D.G. & Radda, G.K. The steady-state rate of ATP synthesis in the perfused rat heart measured by 31P NMR saturation transfer. *Biochem. Biophys. Res. Commun.* **103**, 1052-1059 (1981).
4. Zweier, J.L. & Jacobus, W.E. Substrate-induced alterations of high energy phosphate metabolism and contractile function in the perfused heart. *J. Biol. Chem.* **262**, 8015-8021 (1987).
5. Gaspar, R., Brey, W.S. & Andrew, E.R. P-31 magnetic-relaxation of phosphocreatine in solution *Chem. Phys. Lett.* **184**, 17-20 (1991).
6. Gaspar, R. & Andrew, E.R. P-31 magnetic-relaxation of inorganic orthophosphate in solution *Chem. Phys. Lett.* **170**, 171-174 (1990).
7. Jupin, M. et al. Application of the steady-state variable nutation angle method for faster determinations of long T(1)s-an approach useful for the design of hyperpolarized MR molecular probes. *Magnet. Reson. Insights* **8**, 41-47 (2015).
8. Soto, G.E., Zhu, Z.H., Evelhoch, J.L. & Ackerman, J.J.H. Tumor P-31 NMR pH measurements in vivo: A comparison of inorganic phosphate and intracellular 2-deoxyglucose-6-phosphate as pH(nmr) indicators in murine radiation-induced fibrosarcoma-1. *Magn. Reson. Med.* **36**, 698-704 (1996).
9. Lide, D.R. (ed.) CRC Handbook of Chemistry and Physics p. 1241, Edn. 84. (CRC Press, 2004).

https://archive.org/stream/Handbook_of_Chem_and_Physics_CRC_Press_84th_Ed_2004_WW#page (Accessed July 2017)
